# Iron-driven secondary injury after intracranial hemorrhage: mitochondrial dysfunction and ferroptosis

**DOI:** 10.3389/fnins.2026.1838011

**Published:** 2026-09-03

**Authors:** Maryam Maghareh, Enrique M. Carrera, Dominick Cicala, ASM Roknuzzaman, Rapty Sarker, Werner J. Geldenhuys, Jason D. Huber

**Affiliations:** 1 Department of Pharmaceutical Sciences, School of Pharmacy, West Virginia University, Morgantown, WV, United States; 2 Department of Neuroscience, School of Medicine, West Virginia University, Morgantown, WV, United States

**Keywords:** cerebral ischaemia, hemorrhagic stroke, mitochondrial dysfunction, neuroinflammation, neurovascular unit (NVU), oxidative stress

## Abstract

Intracranial hemorrhage is a devastating neurological condition associated with high mortality and limited therapeutic options beyond surgical management. Increasing evidence identifies iron released from hemoglobin degradation as a central mediator of secondary brain injury and a critical trigger for chronic neurodegenerative processes that persist long after the acute hemorrhagic event across hemorrhage subtypes, including intracerebral, subarachnoid, intraventricular, and subdural hemorrhage. Excess iron drives oxidative stress through reactive oxygen species generation and lipid peroxidation, disrupts blood–brain barrier integrity, and induces profound mitochondrial dysfunction. These pathological processes converge on ferroptosis, an iron-dependent form of regulated cell death characterized by glutathione peroxidase 4 (GPX4) inactivation, lipid peroxidation, and distinctive mitochondrial structural abnormalities. Experimental and clinical studies consistently demonstrate biomarkers of iron overload, oxidative damage, mitochondrial injury, and ferroptotic signaling in hemorrhagic brain injury. Iron-mediated mechanisms also contribute to delayed complications, including neuroinflammation, white matter injury, and delayed cerebral ischemia, as well as progressive cognitive impairment, thereby extending injury beyond the acute phase and promoting sustained neuronal vulnerability. This review explores current evidence linking iron accumulation, mitochondrial dysfunction, and ferroptosis across intracranial hemorrhage subtypes and highlights emerging therapeutic strategies targeting iron chelation, ferroptosis inhibition, enhancement of endogenous antioxidant defenses, and mitochondrial protection. Targeting this interconnected pathological axis represents a promising strategy for the development of disease-modifying therapies aimed at mitigating secondary brain injury and limiting long-term neurobiological remodeling and improving neurological outcomes following intracranial hemorrhage.

## Introduction

Intracranial hemorrhage, characterized by bleeding within the skull, occurs in approximately 40 per 100,000 individuals per year and remains a major global cause of morbidity and mortality (Garton et al., 2016). Rupture of blood vessels can result in significant neurological dysfunction, including impaired speech, visual loss, memory impairment, paralysis, and potential death. Five types of intracranial hemorrhage include: intraventricular hemorrhage (IVH), epidural hemorrhage (EDH), subarachnoid hemorrhage (SAH), intraparenchymal or intracerebral hemorrhage (ICH) and subdural hemorrhage (SDH) (Ahmed and Prakasam, 2025).

While acute clinical management focuses primarily on surgical intervention and stabilization, growing attention has shifted toward the mechanisms underlying secondary brain injury (Chiang et al., 2025). This framing identifies ICH not just as an isolated vascular event, but as a potentiating event for chronic neurodegeneration. Accumulating evidence indicates that secondary brain injury after intracranial hemorrhage is mediated by toxic blood-derived components, with hemoglobin-released iron emerging as a major pathological factor. Following bleeding into the intracranial compartments, erythrocyte lysis and hemoglobin degradation liberate iron ions that catalyze lipid peroxidation, generate reactive oxygen species, and disrupt mitochondrial function, thereby amplifying neuroinflammation and contributing to cellular injury that may persist beyond the acute phase of hemorrhage (Garton et al., 2016; Lok et al., 2011; Shao et al., 2022; Ju and Hang, 2025).

The cellular damage that develops following intracranial hemorrhage is mediated through multiple, often overlapping forms of regulated and unregulated cell death. Apoptosis is a programmed form of cell death characterized by cytoplasmic shrinkage, chromatin condensation, and nuclear fragmentation (Moujalled et al., 2021). In contrast, necrosis is an uncontrolled, proinflammatory cell death marked by cellular swelling and plasma membrane rupture (Rinald and Troy, 2025). Pyroptosis, a regulated inflammatory form of necrotic cell death triggered by cytosolic danger or pathogen-associated signals, also exhibits cellular swelling and membrane disruption well (Broz, 2025). Intracellular homeostasis maintenance can be done through a process called autophagy. In this process organelles and proteins are degraded and recycled. Even though, autophagy’s role is to protect the cells, exacerbated autophagy may contribute to cell death (Liu S. et al., 2023). Among these pathways ferroptosis has emerged as a relevant mechanism in intracranial hemorrhage because it is driven by iron-dependent lipid peroxidation. Ferroptosis is characterized by excessive intracellular iron accumulation, oxidative membrane damage, and impaired antioxidant defenses, particularly dysfunction of lipid peroxide detoxification systems, ultimately resulting in membrane failure and cell death (Li et al., 2020; Zhou et al., 2024). Although iron-dependent oxidative stress contributes to ferroptosis, oxidative stress alone does not constitute direct evidence of ferroptotic cell death, which requires additional molecular or functional validation.

Despite decades of investigation, no FDA-approved therapy effectively mitigates secondary brain injury after hemorrhage, highlighting the urgent need for deeper mechanistic understanding and targeted treatments. Several previous reviews have summarized iron toxicity, ferroptosis, or mitochondrial dysfunction following intracerebral hemorrhage. Rather than proposing a novel mechanistic pathway, in this review, we examine the current evidence linking iron accumulation oxidative stress, mitochondrial dysfunction, and ferroptosis to secondary brain injury across multiple intracranial hemorrhage subtypes including ICH, SAH, IVH, SDH, and EDH. Additionally, we compare subtype-specific mechanisms, discuss cell-type-specific responses, evaluate the emerging therapeutic strategies targeting interconnected pathways and discuss critical knowledge gap that must be addressed to facilitate clinical translation.

## Cell type-specific susceptibility to ferroptosis

The central nervous system (CNS) is comprised of multiple cell types including neurons, pericytes, microglia (glia), astrocytes (glia), endothelial cells, and oligodendrocytes (glia), all which work in tandem to maintain homeostasis and a functional blood–brain barrier (BBB) (Bradl and Lassmann, 2010; Kugler et al., 2021). Despite all these cell types being vulnerable to ferroptosis following intracranial hemorrhage, each cell type contains differing susceptibilities and responses to ferroptosis with neurons, microglia, and endothelial cells having some of the most drastic alterations (Kenny et al., 2019; Fan et al., 2020; Ryan et al., 2023; Park et al., 2021; Liu et al., 2025; Li J. et al., 2022). Neurons are particularly susceptible to ferroptosis due to their large membrane surface area, PUFA membrane composition, increased iron transporters following injury, and upregulation of lipid peroxidizing proteins (Kenny et al., 2019; Jacquemyn et al., 2024; Liang et al., 2022). Dopaminergic neurons demonstrate this susceptibility as they only require minute concentrations of ferroptotic-inducers erastin and RSL3 to exert mitochondrial dysfunction and cell death even when compared to other neuronal cell lines (Tong et al., 2022; Renner et al., 2024). Other work suggests neuronal ferroptosis is closely related to endoplasmic reticulum (ER) stress after ICH as inhibiting the PERK/eIF2α/ATF4/CHOP/GPX4 pathway mitigated ferroptotic damage marked by ROS generation, thereby suggesting ferroptosis in neurons may contain multiple driving organelles following injury (Lu et al., 2024). Microglia, a CNS macrophage, is another cell highly susceptible cell to ferroptosis following injury (Pesämaa et al., 2026; Jiao et al., 2022). Microglia can increase their intracellular accumulation through increased ferroportin transportation which associated with higher levels of lipid peroxidation, decreased reduction system components, and cell death (Jiao et al., 2022). This can cause a cascade of inflammatory signaling as peroxidized lipids can damage mitochondrial DNA, creating strong damage-associated molecular patterns and lead to NLRP3 inflammasome formation, thereby releasing inflammatory cytokines IL-1β and IL-18 which can exaggerate ROS production and disrupt iron hoemostasis (Fawzy et al., 2026; Ren et al., 2026). Microglial ferroptosis has been suggested to drive death in other cell types, exacerbating primary injury (Ryan et al., 2023; Jiao et al., 2022). In primary culture, microglia upregulate *SEC24B*, a component of COPII-dependant cargo transport, in response to ferroptotic stress which subsequently increases ferroptosis in microglia, astrocytes, and neurons. Critically, knock-out of this gene in microglia delays toxicity in astrocytes and neurons, indicating ferroptotic microglia are can regulate damage in other cells (Ryan et al., 2023). Microglial upregulation of *LCN2*, a siderophore-binding protein, drives microglial ferroptosis after ICH. Knockout of this gene mitigates both neuronal and overall cell death (Fei et al., 2024). These studies suggest that aberrant microglial iron regulation not only drives microglial ferroptosis but creates local environments that can drive cell death in surrounding cells. Endothelial cells represent another significant cell vulnerable to ferroptosis and a determinant of cerebrovascular ferroptosis (Liu et al., 2025; Liu Q. et al., 2023; Fang et al., 2022; Qin et al., 2026; Liang et al., 2024). Positioned at the interface between the blood and brain, endothelial cells regulate the passage of ions such as ferrous and ferric iron from crossing the BBB (Qin et al., 2026). Upon damage, ferrous iron accumulation within endothelial cells can lead to ferroptosis in the endothelial cells as discussed prior (Liu Q. et al., 2023; Fang et al., 2022; Liang et al., 2024). However, damage or death of these cells can loosen tight junctions such as claudin and occludin, allowing the passage of iron into the brain to drive ferroptosis in other cells (Fang et al., 2022; Zhang et al., 2026; Hu et al., 2024). Strategies that ameliorate BBB disruption by targeting ferroptotic endothelial cells have been shown to mitigate ferroptosis and other secondary damage in neurodegenerative disease (Saralkar et al., 2020; Vijikumar et al., 2022).

### Intracerebral hemorrhage (ICH)

Intracerebral hemorrhage (ICH) is a highly lethal subtype of stroke, with mortality rates approaching 53–59%. Among many causes, chronic hypertension and cerebral amyloid angiopathy represent the most common etiological factors (Magid-Bernstein et al., 2022; Bautista et al., 2021). In ICH, vessel rupture leads to blood accumulation within the brain parenchyma and initiates two phases of injury: primary and secondary. Primary brain injury develops rapidly and is largely mechanical, driven by hematoma size, mass effect, and tissue compression. The risk of hematoma expansion is greatest within the first 3 h and generally stabilizes after 6 h (Puy et al., 2023).

Secondary injury, in contrast, is more complex and involves a wide range of pathological processes (Fu et al., 2021). A central driver of this secondary phase is iron released from hemoglobin degradation following erythrocyte lysis (Li Z. et al., 2022). Iron progressively accumulates in perihematomal tissue, becoming detectable within 24 h, peaking around day 7, and remaining elevated for at least 14 days (Qing et al., 2009). Excess iron catalyzes the Fenton reaction, leading to sustained production of reactive oxygen species (ROS) and lipid peroxidation, thereby amplifying oxidative damage (Bradl and Lassmann, 2010; Kugler et al., 2021; Kenny et al., 2019). Furthermore, post-hemorrhagic iron deposits and subsequent mitochondrial dysfunction may serve as catalysts for protein misfolding and aggregation, such as tau and amyloid beta, mirroring the pathology of Alzheimer’s disease.

Secondary brain injury in ICH is characterized by BBB disruption, brain edema, neuroinflammation, and progressive neurological dysfunction, which often leads to a persistent state of neuroinflammation in which microglia transition into a chronic neurodegenerative phenotype that contributes to long-term cognitive decline. Among these, mitochondrial dysfunction plays a pivotal role (Chen Y. et al., 2024). Iron-mediated oxidative stress directly impairs mitochondrial integrity, disrupts energy production, and promotes neuronal damage. Because neuronal survival is tightly dependent on mitochondrial homeostasis, mitochondrial injury is a central mechanism underlying neurodegeneration (Gong et al., 2023; Chen W. et al., 2021).

**Figure 1:**
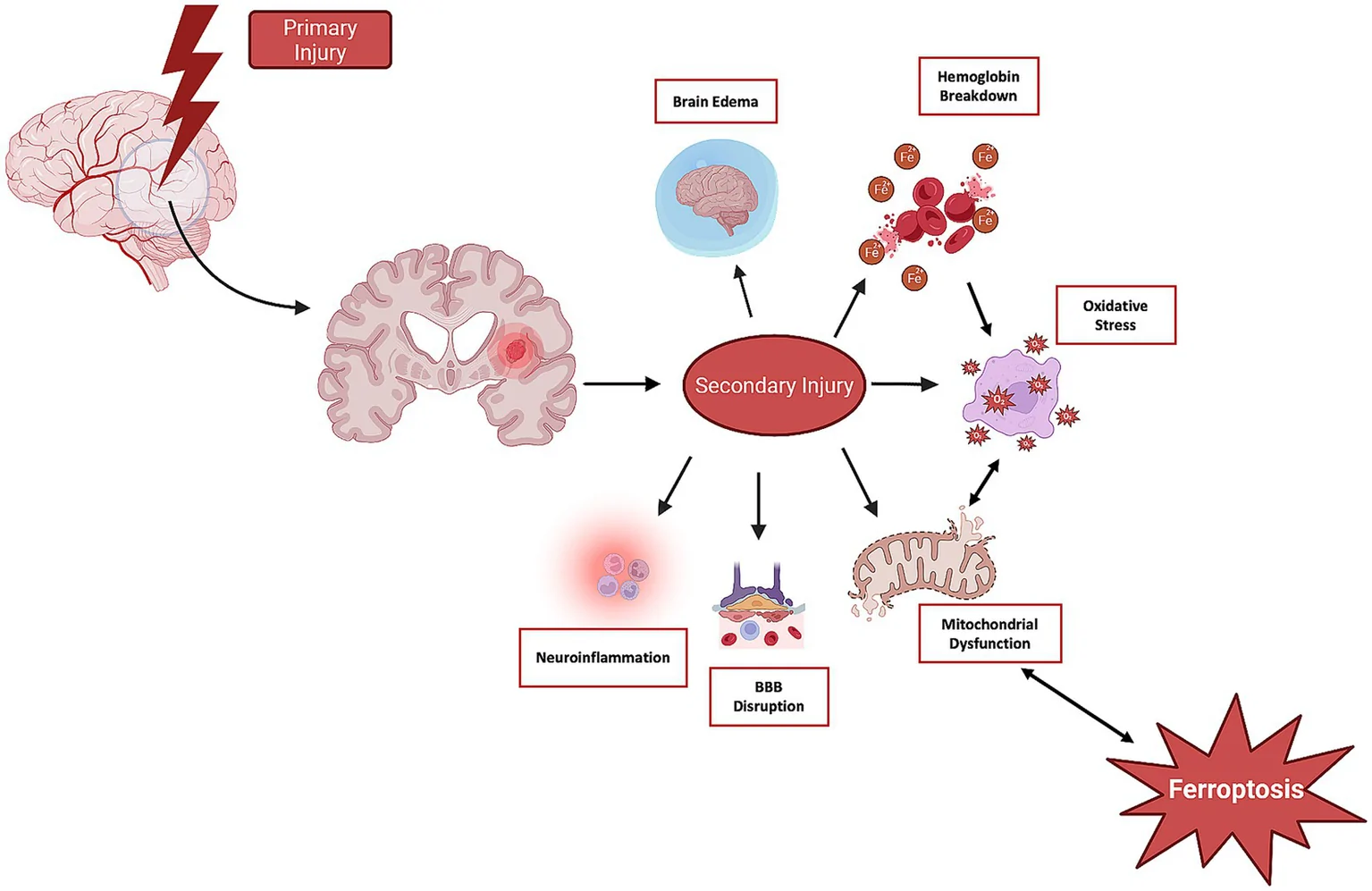
Iron-driven secondary injury mechanisms after intracerebral hemorrhage. Following intracerebral hemorrhage, primary injury initiates a cascade of secondary injury mechanisms, including hemoglobin breakdown, mitochondrial dysfunction, oxidative stress, blood–brain barrier disruption, neuroinflammation, and brain edema. These interconnected processes promote iron-dependent lipid peroxidation and collectively contribute to ferroptosis, which further exacerbates secondary brain injury.

Recent evidence has identified ferroptosis as a key mechanism linking iron overload to neuronal death after ICH. Ferroptosis is characterized by iron-dependent lipid peroxidation, depletion of antioxidant defenses such as glutathione peroxidase 4 (GPX4), and distinct mitochondrial abnormalities, including shrunken morphology and loss of cristae. Proteomic analysis of human ICH tissue reveals pronounced mitochondrial injury alongside ferroptosis-associated changes. Experimental induction of mitochondrial dysfunction, for example, using rotenone, exacerbates ferroptotic neuronal death, further supporting a mechanistic connection between mitochondrial impairment and ferroptosis in ICH (Ryan et al., 2023; Park et al., 2021; Liu et al., 2025; Li J. et al., 2022; Jacquemyn et al., 2024) (Figure 1).

Biochemical evidence from experimental ICH models further supports ferroptotic involvement, including increased levels of Fe^2+,^ ROS, and malondialdehyde (MDA), accompanied by decreased activity of endogenous antioxidant enzymes such as superoxide dismutase (SOD), catalase (CAT), and glutathione peroxidase (GSH-PX). These molecular changes represent hallmark features of ferroptotic injury (Wei et al., 2024). Together, these findings position mitochondrial dysfunction and ferroptosis as interconnected and central mechanisms driving secondary brain injury following ICH (Figure 1).

### Subarachnoid hemorrhage (SAH)

Subarachnoid hemorrhage (SAH), particularly of aneurysmal origin, initiates a complex cascade of secondary injuries, with accumulating preclinical evidence implicating iron toxicity as a major contributor to secondary brain injury (Lee et al., 2010; Li et al., 2021; Fujii et al., 2013; Ward et al., 2014). Following vascular rupture, red blood cells (RBCs) accumulate in the subarachnoid space and undergo lysis, releasing hemoglobin (Hb), which is catabolized to heme and subsequently to ferrous iron (Fe^2+^) via heme oxygenase-1 (HO-1) activity. The level of non-heme iron rises steadily over the initial 72-h period following SAH (Bi et al., 2025). Under physiological conditions, this iron is sequestered by ferritin or exported by ferroportin (Schmidt, 2015; Liu R. et al., 2023). However, after SAH, excess iron can overwhelm endogenous buffering system (Ward et al., 2014; Ganz, 2005; Mancias et al., 2014; Morris et al., 2018), increasing the labile iron pool and promoting oxidative stress (Deng et al., 2023).

In addition to erythrocyte lysis, blood–brain barrier (BBB) disruption further facilitates iron entry into the brain parenchyma. Experimental studies have shown that hemoglobin and its degradation products are directly toxic to the endothelium, inducing lipid peroxidation and inflammatory cascades that contribute to tight junction degradation (Daglas and Adlard, 2018; Frimat et al., 2019; Zhang et al., 2016; Lee et al., 2010; Higashi et al., 2014). Oxyhemoglobin, for example, scavenges nitric oxide (NO), impairing vasodilatory function and initiating vasospasm (Pluta, 2005; Asano, 1999). Consistent with enhanced iron accumulation, experimental studies have reported increased ferritin and transferrin receptor (TfR1) expression in perivascular astrocytes and neurons following SAH (Ge et al., 2024; Jamrus et al., 2025; Morris et al., 2018; Li et al., 2011; Tan et al., 2016).

Mitochondria are particularly vulnerable to this iron-induced oxidative stress. Excess Fe^2+^ catalyzes the Fenton reaction with hydrogen peroxide (H_2_O_2_), generating hydroxyl radicals (•OH) that damage mitochondrial DNA, proteins, and lipid membranes (Yan et al., 2022; Zhao, 2019). Electron microscopy in animal models of SAH reveals swollen, dysmorphic mitochondria with disrupted cristae, consistent with oxidative injury (Yan et al., 2015). Experimental evidence suggests that impaired mitochondrial function contributes to ATP depletion, calcium dysregulation, and activation of pro-apoptotic signaling (Helbok et al., 2015; Jacobsen et al., 2014; Dai et al., 2024). Furthermore, mitochondrial ROS may amplify oxidative stress, creating a feed-forward cycle of cellular injury (Cui et al., 2021; Rose et al., 2017; Zhang and Zhang, 2024; Zhou et al., 2023).

**Figure 2:**
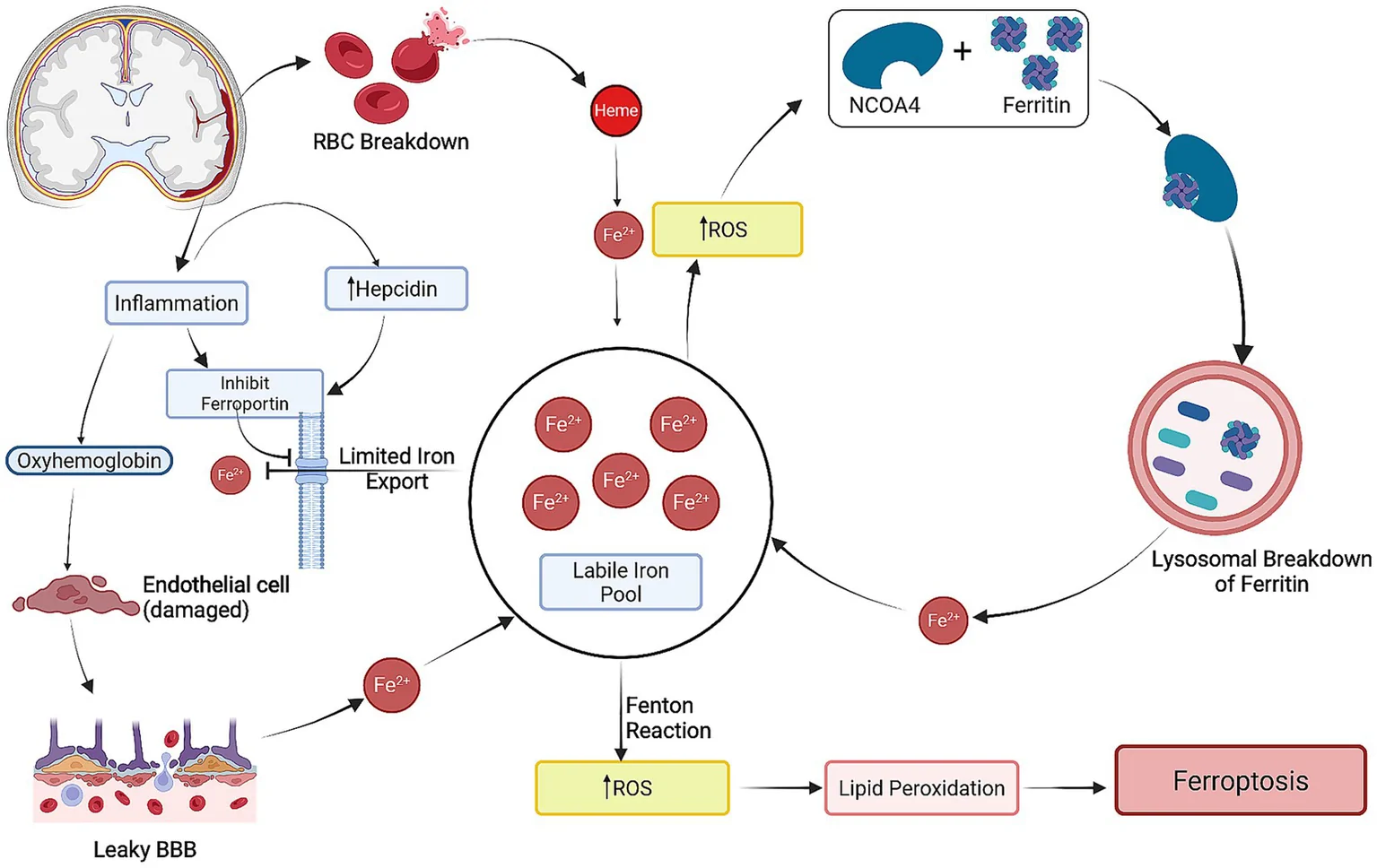
Mechanistic overview of iron-mediated injury after subarachnoid hemorrhage. Subarachnoid bleeding leads to erythrocyte breakdown and accumulation of hemoglobin-derived iron in the subarachnoid space. Elevated labile iron directly increases ROS production and, through Fenton chemistry, promotes lipid peroxidation. Hepcidin-mediated inhibition of ferroportin further promotes intracellular iron accumulation by limiting iron export, while NCOA4 (nuclear receptor coactivator-4)-mediated ferritinophagy releases ferritin-bound iron pool. These oxidative events impair mitochondrial function and activate ferroptotic pathways, contributing to neuronal injury and secondary brain damage.

Accumulating experimental evidence identifies ferroptosis as a key pathway contributing to iron-induced neurodegeneration following SAH (Deng et al., 2023; Li et al., 2021; Dixon et al., 2012; Stockwell et al., 2017). In experimental SAH, glutathione (GSH) depletion and downregulation of GPX4 have been observed in cortical neurons, coinciding with increased malondialdehyde (MDA) and 4-hydroxynonenal (4-HNE) levels, hallmarks of lipid peroxidation (Feng et al., 2023; Zhang W. et al., 2024). In preclinical SAH models, pharmacological inhibition of ferroptosis using agents such as ferrostatin-1 (Fer-1) or liproxstatin-1 (Lip-1) reduces lipid peroxidation, preserves GPX4 activity, and improves neurological outcomes (Cao Y. et al., 2021; Liu et al., 2022; Gao et al., 2020; Yuan et al., 2022; Chen J. et al., 2024; Cao et al., 2023). Similarly, experimental overexpression of GPX4 and treatment with iron chelators such as deferoxamine (DFO) have been shown to confer neuroprotection and preserve mitochondrial integrity in experimental models (Gao et al., 2020; Jia et al., 2023) (Figure 2).

Recent studies have further implicated ferritinophagy, the autophagic degradation of ferritin mediated by NCOA4, in SAH-induced ferroptosis. This process liberates stored iron, exacerbating ROS production (Yu et al., 2024). Inhibiting NCOA4 or general autophagy suppressed ferroptosis markers and improved BBB integrity in rodent SAH models (Fang et al., 2021). Moreover, emerging evidence indicates that the effects of the hepcidin–ferroportin axis are cell type-dependent (You et al., 2022). Astrocyte-derived hepcidin regulates ferroportin expressed on endothelial cells, neurons, and glial cells (You et al., 2022; Zhang et al., 2020; Mezzanotte et al., 2022; Vela, 2018). At the BBB, hepcidin-mediated ferroportin degradation limits pathological iron transport into the brain parenchyma and supports BBB integrity (You et al., 2022; Mezzanotte et al., 2022; Ginzburg, 2019; Wu et al., 2023). Conversely, prolonged ferroportin suppression within neural cells promotes intracellular iron retention, expands the labile iron pool, and enhances ferroptotic injury (Bao et al., 2021; Nolt and Connor, 2024; Tang et al., 2020).

The consequences of iron-mediated damage extend beyond early brain injury and contribute significantly to delayed cerebral ischemia (DCI), a leading cause of morbidity after SAH (Eren et al., 2022). Traditionally attributed to vasospasm, DCI is now understood as a multifactorial process involving microvascular dysfunction, inflammation, and metabolic crisis (Eren et al., 2022; Francoeur and Mayer, 2016). Iron catalyzes endothelial injury and vasoconstriction through ROS generation and NO depletion. Free iron and hemoglobin products in the CSF correlate with the severity of vasospasm in patients, and iron chelation reduces vasospasm and infarct burden in preclinical studies (Bi et al., 2025). At the microvascular level, iron-induced oxidative stress disrupts endothelial metabolism and promotes leukocyte adhesion, further impairing perfusion (Scioli et al., 2020). Neurons already stressed by iron overload and mitochondrial dysfunction are more susceptible to hypoxia during DCI episodes (Weinberg et al., 2000). Collectively, these findings suggest that iron dysregulation can be a unifying contributor to both early and delayed pathological processes following SAH.

### Intraventricular hemorrhage (IVH)

Intraventricular hemorrhage is a form of hemorrhagic stroke in which bleeding occurs within the ventricular system and frequently arises as a complication of ICH or SAH (Zhang et al., 2017). Although IVH rarely develops in adults without underlying pathology, it represents a major clinical problem in preterm infants due to their undeveloped germinal matrix and is strongly associated with the development of posthemorrhagic hydrocephalous (Apeksha Reddy et al., 2023). Initiation of ferroptosis in IVH begins through the lysis of erythrocytes in the ventricles followed by iron release and generation of ROS (Apeksha Reddy et al., 2023; Wang H. et al., 2025). While relatively few studies have directly examined ferroptosis in IVH, accumulating evidence supports a pathological role for iron-dependent cell death and suggests therapeutic potential for ferroptosis-targeting strategies, particularly in neonatal disease.

Mechanistically, hemoglobin breakdown products within the ventricular system directly impair mitochondrial function. In oligodendrocyte progenitor cells, iron-induced oxidative stress leads to loss of mitochondrial membrane potential and reduced ATP production, providing a mechanistic explanation for the vulnerability of periventricular white matter after IVH. Consistent with this, *in vivo* studies demonstrate that iron deposition peaks between 3 and 7 days after IVH, coinciding with oligodendrocyte loss and increased microglial activation. Transcriptomic analyses reveal enrichment of ferroptosis-related pathways in oligodendrocytes, and treatment with the ferroptosis inhibitor Fer-1 improves oligodendrocyte survival and myelin integrity. Additional experimental evidence implicates complement-mediated erythrocyte lysis in iron accumulation, as inhibition of membrane attack complex formation reduces iron deposition and improves neurological outcomes. Iron chelation using deferoxamine similarly decreases ventricular iron burden and ameliorates posthemorrhagic complications. These experimental findings are further supported by RNA-sequencing data from preterm infants with IVH, which identify ferroptosis, fatty acid metabolism, and mitophagy as among the most upregulated pathways (Wei et al., 2024; Lee et al., 2010; Li et al., 2021; Fujii et al., 2013; Ward et al., 2014).

Beyond local ventricular damage, pericytes have increasingly been implicated as an important determinant of IVH outcomes through the prevention of aberrant angiogenesis into the ventricles (Ballabh, 2010; Vinukonda et al., 2010). Loss of TGF-b signaling in pericytes leads to BBB breakdown in the germinal matrix by failing to repress angiopoietin-2, a promoter of angiogenesis (Dave et al., 2024). Another study found that injection of betamethasone in preterm rabbit pups led to increased pericyte coverage in the germinal matrix, which was associated with a decrease in TUNEL-positive cells, indicating pericyte coverage reduced IVH (Vinukonda et al., 2010). A recent report on the cerebrospinal fluid (CSF) composition of infants with IVH found that CSF could induce ferroptosis in choroid plexus epithelial cells and pericytes. Intriguingly, this was tightly correlated with the presence of chondroitin sulfate proteoglycan 4 (CSPG4), a known marker of pericytes responsible for cell-adhesion (Schiffer et al., 2018). Genetic disruption of CSPG4 restores GPX4 expression and reduces ferroptotic signaling *in vivo*, suggesting a functional contribution of pericytes to ferroptosis-related injury (Chen et al., 2025).

**Figure 3:**
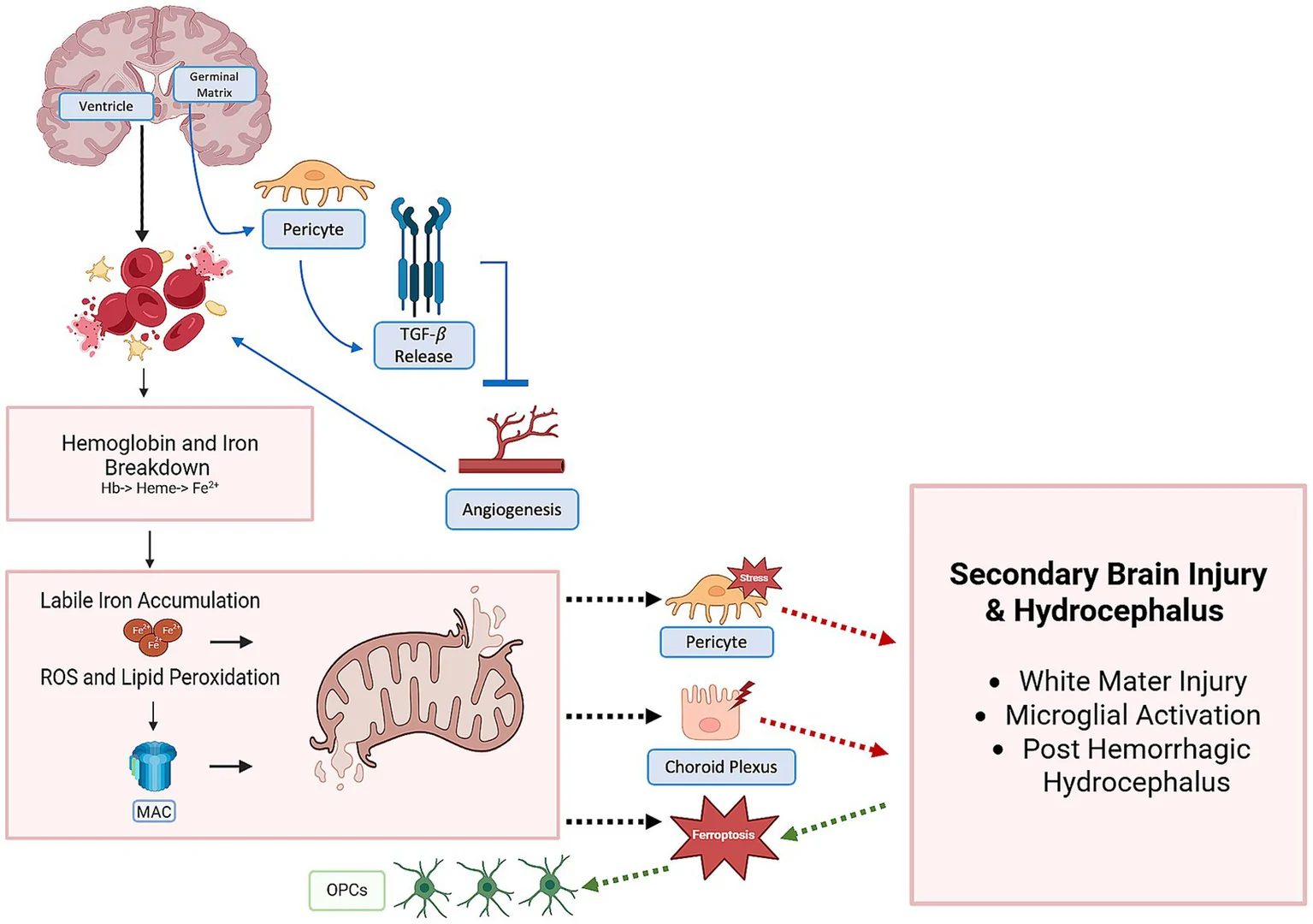
Mechanism of iron-dependent mitochondrial dysfunction and ferroptosis after intraventricular hemorrhage. IVH results in erythrocyte lysis and hemoglobin breakdown, releasing heme and free iron into the ventricular system. Iron accumulation promotes ROS generation, lipid peroxidation, and mitochondrial dysfunction, contributing to ferroptotic cell death. These events are associated with injury to oligodendrocyte progenitor cells (OPCs), pericyte, and the choroid plexus, as well as angiogenic signaling mediated by transforming growth factor *β* (TGF-β). Emerging evidence suggests that these pathological processes may contribute to white matter injury, microglial activation, and post-hemorrhagic hydrocephalus. Solid arrows indicate established mechanisms: Dashed arrows indicate proposed pathways.

Microglia have also been suggested to contribute to ferroptosis in models of IVH. In spinal cord injury models with IVH, a microglia subcluster was found to highly express ferroptotic genes that could increase ferroptosis in the ventricular system (Wang S. et al., 2025). Experimental studies further suggest that microglia interactions with astrocytes lead to hydrocephalus after IVH (Meng et al., 2023; Tang et al., 2022). Pharmacological inhibition of microglial activation, including suppression of NLRP3 inflammasome signaling, reduces edema, limits secondary injury, and attenuates downstream glial activation (Tang et al., 2022; Liddell et al., 2024). Collectively, these findings underscore the importance of coordinated interactions among oligodendrocytes, pericytes, and microglia in shaping ferroptosis-related pathology after IVH (Figure 3).

### Subdural hematoma (SDH)

Subdural hematoma (SDH) results from bleeding beneath the dura mater and is characterized by blood accumulation that exerts mechanical compression on cortical tissue while also initiating a cascade of molecular and cellular injury responses. The populations most at risk are infants and older adults. In infants, SDH frequently arises from rupture of bridging veins during abusive head trauma, whereas in older adults, it is commonly associated with falls and the use of anticoagulant medications. Clinically, SDH is classified as acute or chronic based on the temporal evolution and pathological features of the hematoma (Pierre and Kondamudi, 2025; Baechli et al., 2004).

Acute SDH typically develops within 72 h of trauma and is associated with rapid bleeding, mass effect, and risk of herniation. Chronic SDH evolves more slowly over weeks and is pathologically distinct due to the formation of vascularized neomembranes surrounding the hematoma (Arunachalam Sakthiyendran et al., 2025; Edlmann et al., 2017; Stanisic et al., 2012). These membranes exhibit high angiogenic activity, driven in part by vascular endothelial growth factor (VEGF), which promotes the development of immature, leaky microvessels and contributes to repeated microhemorrhage. In parallel, sustained inflammatory signaling further perpetuates hematoma expansion. Pro-inflammatory cytokines such as IL-6 and IL-8 enhance vascular permeability and stimulate matrix metalloprotease activity, creating a self-perpetuating cycle of inflammation, angiogenesis, and membrane fragility. These processes help explain why chronic SDH often persists or recurs despite relatively modest initial injury (Pluta, 2005; Ge et al., 2024; Jamrus et al., 2025; Morris et al., 2018; Li et al., 2011).

SDH has been consistently associated with mitochondrial abnormalities (Verweij et al., 2000). Electron microscopy of post-SDH tissue has revealed swollen mitochondria (Ishikawa et al., 2009), a hallmark of disrupted ionic and osmotic homeostasis. The mechanism by which the mitochondrial swelling is induced should remain an area of future research. Although the precise upstream triggers remain unclear, mitochondrial swelling is believed to result from prolonged opening of the mitochondrial permeability transition pore (mPTP), leading to uncontrolled influx of solutes and water into the organelle (Zhao, 2019; Yan et al., 2015; Helbok et al., 2015). Swelling of mitochondria induces a stretching/remodeling of the cristae, impairing ATP synthase dimerization and reducing ATP production (Jacobsen et al., 2014; Dai et al., 2024; Cui et al., 2021). If enough swelling and cristae remodeling occur, the outer mitochondrial membrane fragments, leading to the release of pro-apoptotic factors and eventual cell death (Olichon et al., 2003; Zick et al., 2009). Although these mechanisms are biologically plausible, further research is required to confirm that SDH is operating via the same mechanism.

Functional studies further support a causal role for mitochondrial impairment in SDH pathology. In experimental models of acute SDH, pharmacological activation of mitochondrial ATP-sensitive potassium (mitoKATP) channels confers neuroprotection. These channels regulate mitochondrial membrane potential, calcium handling, and ROS production, underscoring the importance of mitochondrial stability in determining cellular survival after subdural blood exposure. Complementing these experimental findings, metabolomic analyses of chronic SDH fluid from human patients reveal alterations in lipid metabolism and reduced acylcarnitine levels, consistent with impaired mitochondrial fatty acid b-oxidation and disrupted bioenergetics, particularly in cases prone to recurrence. Collectively, these data support a model in which SDH involves mitochondrial bioenergetic failure, altered redox regulation, and heightened vulnerability to metabolic stress (Nakagawa et al., 2013; Kipele et al., 2023), however whether mitochondrial impairment is the primary cause of the pathologic changes following TBI or is a secondary response to tissue injury remains unclear.

**Figure 4:**
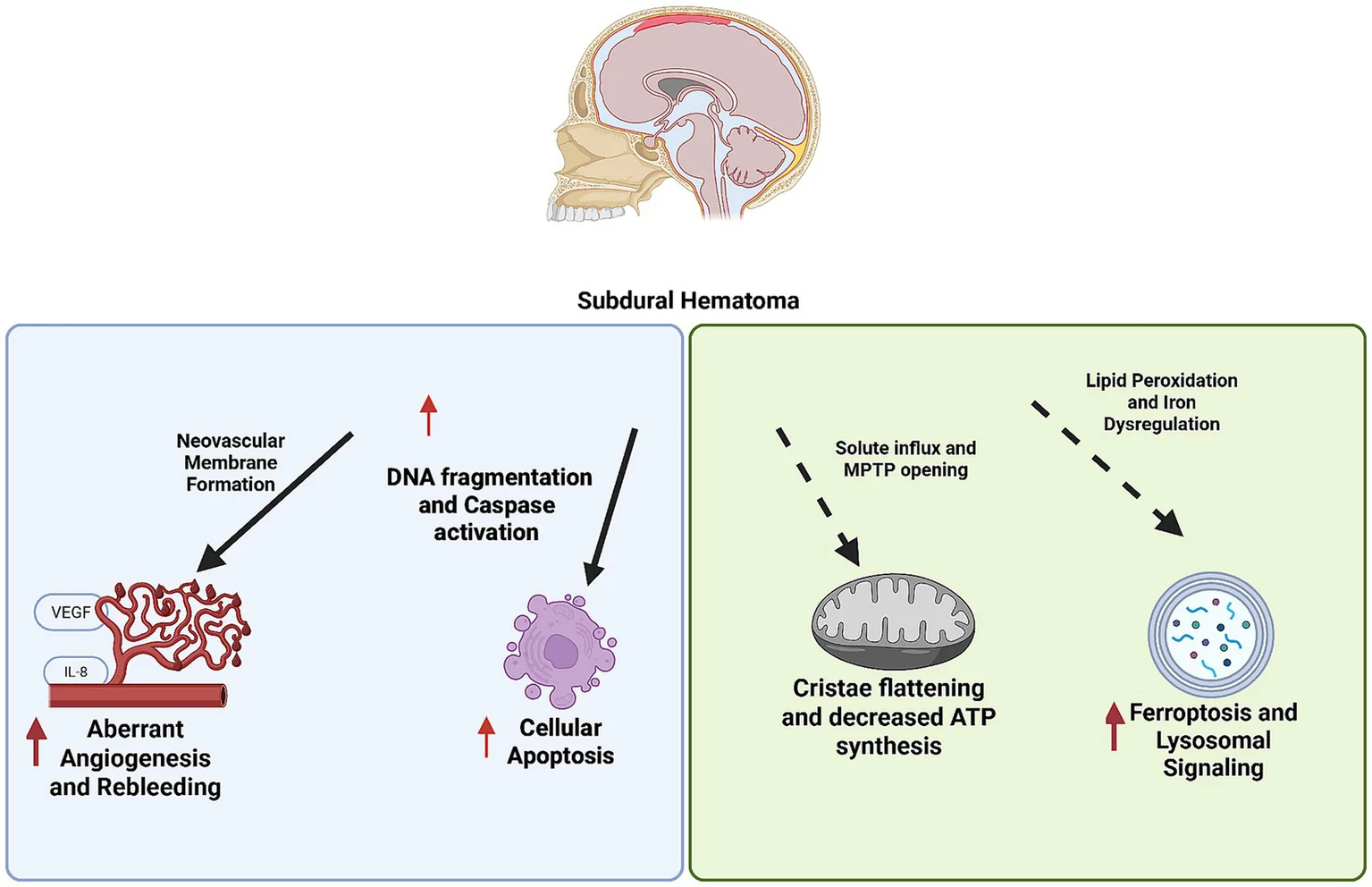
Mitochondrial dysfunction in subdural hematoma. Subdural hematoma is associated with ultrastructural mitochondrial abnormalities in affected tissue, including mitochondrial swelling, cristae remodeling, and impaired bioenergetic function. These changes are consistent with dysregulation of. Mitochondrial permeability contributes to reduced ATP production and increased susceptibility to cell death. Emerging transcriptomic evidence further suggests that oxidative stress and iron dysregulation within the hematoma membrane may engage ferroptosis-related pathways.

While apoptosis and necrosis have long been implicated in SDH pathophysiology, ferroptosis has only recently entered investigation. Existing animal models of SDH replicate many pathological features such as inflammation, intracranial pressure elevation, and white matter injury (Dixon et al., 2012; Stockwell et al., 2017; Zhang W. et al., 2024; Cao Y. et al., 2021). Ferroptosis-specific analyses in these systems, however, remain limited. Recent transcriptomic data from human chronic SDH samples provide preliminary support for ferroptotic involvement. Single-cell RNA sequencing identified upregulation of ferroptosis- and lysosome-related genes within M2-polarized macrophages residing in the hematoma membrane (Zhang Q. et al., 2024). These findings suggest that lipid peroxidation and iron dysregulation may contribute to hematoma persistence or membrane remodeling. Whether this is a driver of cellular injury or a downstream consequence of oxidative stress remains an open question for future studies (Figure 4).

### Epidural hematoma (EDH)

Unlike other types of hemorrhage, Epidural Hematoma (EDH) is primarily a mechanically driven injury and is often considered a surgical emergency with favorable outcomes when promptly treated. EDH is defined by bleeding that accumulates between the skull and the dura mater, rather than between the dura and neural tissue as in subdural hematoma. EDHs most frequently occur among 20-30-year-old males and are rare in the elderly, likely due to the dura mater becoming more adherent to the skull (Khairat et al., 2025). Experimental evidence suggests that both structural compression and exposure to blood products contribute to oxidative and mitochondrial injury in EDH. In a bead-compression model, induction of EDH led to a significant reduction in the density of thalamocortical afferent (TCA) fibers and their dendrites for months after the initial injury. Electron microscopy revealed condensed and darkened mitochondria within affected neurons, consistent with mitochondrial stress. Concurrently, oxidative stress markers such as 3-nitrotyrosine (3-NT) and 4-hydroxynonenal (4-HNE) were significantly elevated, indicating enhanced protein nitration and lipid peroxidation (Yeh et al., 2017). In an autologous-blood injection model, EDH induction was associated with elevated inflammatory cytokines and increased vascular permeability, coinciding with reduced expression of high-mobility group box 1 (HMGB1), a non-histone DNA-binding protein involved in chromatin stability and immune signaling (Gao et al., 2024). Taken together, these findings suggest that both mechanical and molecular stressors synergize to induce mitochondrial dysfunction and oxidative damage in the tissue surrounding the hematoma.

## Long-term neurological sequelae of ICH

Although ICH, SAH, SDH, and IVH differ in anatomical distribution and acute presentation, their long-term pathological trajectories converge through shared iron-driven and vascular mechanisms. Across subtypes, degradation of extravasated blood results in sustained iron accumulation, oxidative stress, and mitochondrial dysfunction that extend beyond the initial hemorrhagic event. In ICH and SAH, persistent iron deposition promotes lipid peroxidation, ferroptotic signaling, and chronic glial activation, creating a maladaptive redox and inflammatory microenvironment (Ju and Hang, 2025; Gao et al., 2020; Yuan et al., 2022; Chen J. et al., 2024). In SDH, prolonged perfusion deficits, impaired glymphatic clearance, and cortical re-expansion failure contribute to sustained metabolic stress and progressive cortical thinning (Nouri et al., 2021; Bin Zahid et al., 2018). Similarly, IVH disrupts cerebrospinal fluid dynamics and exposes periventricular structures to blood breakdown products, predisposing to white matter injury and ongoing inflammatory signaling (Park, 2023). Despite anatomical differences, these shared disturbances in iron homeostasis, neuroinflammation, and cerebrovascular regulation collectively establish a state of prolonged neuronal vulnerability.

Clinically, these sustained molecular and structural alterations translate into long-term neurological and cognitive impairment across hemorrhage subtypes. Survivors of ICH and SAH frequently demonstrate chronic cognitive decline, white matter degeneration, and brain atrophy months to years after the acute event, with emerging evidence linking remote iron deposition to impaired neurogenesis and hippocampal dysfunction (Ju and Hang, 2025; Zhang X. et al., 2024; Aydin and Peker, 2025; Al-Khindi et al., 2010). Chronic SDH is associated with progressive cortical atrophy and dementia-like symptoms, reflecting persistent perfusion dysregulation and structural remodeling (Bin Zahid et al., 2018; Sahyouni et al., 2017). In IVH, especially in vulnerable populations such as preterm infants, ventricular injury and periventricular white matter damage contribute to enduring neurodevelopmental and functional deficits (Park, 2023; Haldrup et al., 2023). Collectively, these observations support a growing paradigm in which intracranial hemorrhage initiates not only acute injury but also sustained neurobiological processes that resemble elements of progressive neurodegenerative pathology.

## Therapeutic opportunities in iron and mitochondria-driven injury

Following intracranial hemorrhage, hemoglobin degradation releases labile iron that catalyzes reactive oxygen species (ROS) formation and drives ferroptotic and mitochondrial injury. Because these events constitute a common axis of secondary brain damage, therapeutic strategies have largely focused on neutralizing iron toxicity, blocking ferroptotic lipid peroxidation, and protecting mitochondria (Feng et al., 2023; Mao et al., 2025). Although these interventions show considerable promise in preclinical models of intracranial hemorrhage, successful clinical translation remains limited by challenges including blood–brain barrier delivery, therapeutic time windows, dosing constraints, and inconsistent efficacy across clinical studies (Liu et al., 2026; Zheng et al., 2025).

A primary strategy involves removing excess redox-active iron before it amplifies oxidative stress. Deferoxamine (DFO), a high-affinity ferric iron (Fe^3+^) chelator, limit iron-catalyzed redox cycling which leads to reduction in Fenton reactions (Fe^2+^ + H_2_O_2_ → Fe^3+^ + •OH) and decreased hydroxyl radical formation. In preclinical models of intracerebral hemorrhage, DFO reduces lipid peroxidation, inflammation, and neuronal injury (Xie et al., 2025; Pawlaczyk and Schroeder, 2021). Across experimental models of ICH and SAH, DFO consistently lowered free iron levels, reduced perihematomal edema, preserved blood–brain barrier integrity, and improved neurological outcomes (Li Z. et al., 2022; Li Y. et al., 2017). These encouraging preclinical findings have led to several clinical studies, with deferoxamine representing the most extensively investigated iron-chelating therapy for intracerebral hemorrhage (Selim et al., 2019; Yeatts et al., 2013). Although clinical trials have demonstrated favorable effects on perihematomal edema and hematoma resolution, improvements in long-term neurological outcomes have been inconsistent (Selim et al., 2019; Sun T. et al., 2023; Yu et al., 2015). Meta-analyses and *post hoc* analyses of the phase II i-DEF trial suggest that differences in treatment timing, dosing strategies, patient selection, and methods of outcome assessment may contribute to the variable clinical findings, highlighting the need for further optimization before routine clinical implementation (Selim et al., 2019; Yeatts et al., 2013; Sun T. et al., 2023; Foster et al., 2022). Other iron-targeting therapies have shown promising but predominantly preclinical efficacy through complementary mechanisms. Among these agents, clioquinol reduces oxidative stress and brain swelling (Zhang Y. et al., 2022), minocycline limits iron accumulation and neuroinflammation (Chen J. et al., 2024), while lactoferrin enhances endogenous iron sequestration and mitigates ferroptotic injury in experimental models (Sinopoli et al., 2022; Guan et al., 2024). Collectively, these findings support iron chelation as a promising strategy to attenuate secondary injury after intracranial hemorrhage. However, most evidence remains preclinical, and optimizing drug delivery, therapeutic windows, and patient selection will be essential for successful clinical translation (Liu et al., 2026; Zheng et al., 2025; Foster et al., 2022).

As iron overload promotes ferroptotic lipid peroxidation, therapies targeting ferroptosis represent a complementary strategy to iron chelation. Ferroptosis arises from iron-driven lipid peroxidation and failure of antioxidant defenses, especially glutathione peroxidase 4 (GPX4) (Chen L. et al., 2021). Small-molecule inhibitors such as Fer-1 and Lip-1 act as radical-trapping antioxidants that interrupt lipid peroxidation (Tong et al., 2023; Miotto et al., 2019). In ICH and SAH models, Fer-1 reduced neuronal loss, iron deposition, and ROS, and preserved neurological function (Li Q. et al., 2017; Wang et al., 2023). Additional ferroptosis modulators strengthen endogenous antioxidant defenses through distinct but complementary mechanisms. Coenzyme Q10 (CoQ10), part of the mitochondrial FSP1–CoQ antioxidant system, quenches lipid radicals and attenuates ferroptotic damage (Peng et al., 2024; Rizzardi et al., 2021). Selenium supplementation restores GPX4 activity and ferroptosis resistance (Zheng et al., 2024). Natural compounds such as baicalin and curcumin nanoparticles elevate glutathione and maintain redox balance (Cao et al., 2011; González-Reyes et al., 2013). Activation of Nrf2 further upregulates antioxidant and iron-storage genes (González-Reyes et al., 2013; Rahban et al., 2020), while inhibitors of 5-lipoxygenase (5-LOX) or 12/15-LOX reduce production of oxidized lipids that drive ferroptosis (Ren et al., 2020; Cakir-Aktas et al., 2023). However, most ferroptosis-targeted therapies remain preclinical, and their clinical translation is limited by pharmacokinetic instability, blood–brain barrier delivery, tissue specificity, dosing uncertainty, and the lack of validated biomarkers to monitor ferroptosis inhibition in patients (Liu et al., 2026; Zheng et al., 2025; Duo et al., 2025). Collectively, these approaches support ferroptosis inhibition as a promising preclinical strategy to preserve membrane integrity and limit iron-driven redox collapse after intracranial hemorrhage (Zheng et al., 2025; Duo et al., 2025).

Mitochondria act as both sources and targets of iron toxicity. Excess mitochondrial iron disrupts electron transport, elevates ROS, and depletes ATP (Zhao et al., 2024). Mitochondria-targeted antioxidants, including SS-31 (elamipretide), which binds cardiolipin in the inner mitochondrial membrane, directly reduce mitochondrial ROS and preserve mitochondrial bioenergetics. SS31 restored redox balance, increased mitochondrial DNA copy number, and improved behavioral recovery (Liu et al., 2021; Reddy et al., 2018; Campbell et al., 2019). Other antioxidants, including astaxanthin and melatonin, preserve mitochondrial integrity through distinct mechanisms: astaxanthin inhibits cytochrome-c release and the Bax/Bcl-2 apoptotic axis (Song et al., 2014), while melatonin scavenges free radicals and blocks the mitochondrial permeability transition pore by modulating cyclophilin D (Agil et al., 2021; Andrabi et al., 2004). Regulation of mitochondrial dynamics adds another layer of protection; excessive fission via Drp1 worsens dysfunction, whereas promoting fusion or inhibiting Drp1, such as with intranasal oxytocin, improves survival (Sharp et al., 2015). Enhancing mitophagy through PINK1–Parkin or AMPK activation helps remove damaged mitochondria, reducing ROS leakage and sustaining bioenergetic function (Cao S. et al., 2021). Despite these encouraging experimental findings, most mitochondria-targeted therapies have not advanced beyond preclinical evaluation for intracranial hemorrhage. Their clinical translation remains constrained by challenges including efficient brain delivery, uncertain therapeutic windows, limited pharmacokinetic data, and the need to preserve physiological mitochondrial function while preventing pathological oxidative injury (Liu et al., 2026; Zheng et al., 2025; Tung et al., 2025). Together, these strategies demonstrate that preservation of mitochondrial homeostasis is a promising therapeutic approach, although further studies are needed to establish their safety, efficacy, and clinical applicability following intracranial hemorrhage (Zheng et al., 2025; Tung et al., 2025).

Although mechanistically distinct, iron chelation, ferroptosis inhibition, and mitochondrial protection converge on the same oxidative network. Integrating these complementary modalities may yield synergistic therapeutic effects. For instance, combining an iron chelator with a lipid-peroxidation inhibitor can simultaneously block upstream iron catalysis and downstream membrane damage. Nanotechnology now enables multi-targeted systems; a macromolecular nanoscavenger carrying DFO for iron binding and catechol groups for ROS quenching effectively reduced oxidative injury and improved outcomes in ICH models (Zhu et al., 2021). However, these combination approaches remain largely confined to experimental models, and their successful clinical translation will require optimization of drug delivery, treatment timing, safety profiles, and validation in well-designed clinical studies (Liu et al., 2026; Zheng et al., 2025). Such approaches highlight the therapeutic potential of concurrently targeting iron, ROS, and mitochondria to interrupt the vicious cycle of secondary brain injury. Targeting the interconnected pathways of iron overload, ferroptosis, and mitochondrial dysfunction provides a mechanistically rational approach to limit post-hemorrhagic neurodegeneration. By restoring iron homeostasis, reinforcing antioxidant defenses, and preserving mitochondrial vitality, these therapies address both the triggers and executors of secondary injury. Continued advances in targeted drug delivery, biomarker-guided patient selection, and rational combination strategies may facilitate successful clinical translation and ultimately achieve durable neurovascular protection after intracranial hemorrhage (Liu et al., 2026; Zheng et al., 2025; Duo et al., 2025) (Table 1).

**Table 1:** Summary of therapeutic strategies targeting iron-driven oxidative injury, ferroptosis, and mitochondrial dysfunction after intracranial hemorrhage.

Therapeutic category	Primary mechanism	Representative molecular targets/pathways	Evidence and clinical development	Major translational challenges
Iron chelation	Removes labile Fe^2+^, preventing Fenton chemistry and ROS generation	Iron sequestration ↓ Fe^2+^-driven OH production ↓ lipid peroxidation	Preclinical and Phase II (DFO)	BBB delivery; dosing; therapeutic window; variable clinical efficacy
Direct ferroptosis inhibition	Radical-trapping antioxidants that block lipid peroxidation	Inhibition of phospholipid-ROS chain reactions Protection of PUFA-PEs	Preclinical and experimental	Poor pharmacokinetics; BBB penetration; lack of biomarkers
Enhancing endogenous antioxidant defenses	Strengthens intrinsic redox buffering and boosts GPX4-dependent detoxification	GPX4 activity restoration ↑ GSH synthesis ↑ Nrf2-regulated antioxidant/iron-storage genes	Preclinical and experimental	Target specificity; limited clinical validation
Lipoxygenase (LOX) pathway inhibition	Blocks enzymatic oxidation of PUFAs that drive ferroptosis	↓ Hydroperoxy-PUFAs ↓ LOX-derived oxidized lipids	Preclinical and experimental	Limited efficacy data; no clinical studies
Mitochondrial antioxidants	Neutralizes mitochondrial ROS, stabilizes membranes, protects ETC	Cardiolipin binding (SS31) Which protects cardiolipin from peroxidation and optimizes mitochondrial function.	Preclinical and experimental	Brain delivery; therapeutic window
Regulation of mitochondrial dynamics	Limits pathological fission and supports mitochondrial network integrity	↓ Drp1-mediated fission ↑ Fusion machinery	Preclinical and experimental	Off-target effects; limited translational evidence
Mitophagy enhancement	Removes damaged mitochondria before ROS accumulation worsens injury	↑ Mitophagy flux Removal of dysfunctional mitochondria	Preclinical and experimental	Limited pharmacological modulators; no clinical validation

The table outlines major therapeutic categories, including iron chelation, direct ferroptosis inhibition, enhancement of endogenous antioxidant defenses, lipoxygenase inhibition, mitochondrial antioxidants, modulation of mitochondrial dynamics, and promotion of mitophagy. In addition to the principal mechanisms and molecular targets, the table summarizes the current level of evidence, stage of therapeutic development, and key translational challenges for each therapeutic strategy.

## Translational and clinical evidence

### Treatments targeting iron management

Among the therapeutic opportunities discussed, clinical trials such as The High-Dose Deferoxamine (HI-DEF), a prospective, open label, dose escalation phase I clinical trial evaluated the safety and tolerability of intravenous deferoxamine in patients with spontaneous ICH. This study could establish the safe doing regimen and determined the dose-limiting pulmonary toxicity dose which was priorly the identified as the maximum dose tolerated (62 mg/kg/day for 5 consecutive days) (Anonymous, 2019). Later on, i-DEF, a prospective, multi center, double-blinded, randomized, placebo-controlled, phase II clinical trial explored treatment of brain hemorrhage with this iron chelator, Deferoxamine Mesylate. This trial showed that the drug was safe and well-tolerated at a reduced dose of 32 mg/kg/day IV (3 consecutive days), but at 90 days there was no significant difference between the group that received placebo compared to the group that received deferoxamine (Selim et al., 2019). Although this study did not demonstrate a significant improvement in the primary 90-day functional outcome, a subsequent post-hoc study suggested that deferoxamine may favorably alter the trajectory of neurological recovery up to 180 days post-ICH (Foster et al., 2022).

In order to investigate the translational potential of iron chelation beyond ICH, an ongoing phase II, randomized trial, DISH, evaluates deferoxamine in aneurysmal subarachnoid hemorrhage patients. The purpose of this trial is to examine if deferoxamine can mitigate secondary brain injury and improve neurological outcomes through chelation of free iron which is released through hemoglobin degradation (Pandey, 2025).

### Treatments proposed to influence ferroptosis

Minocycline has attracted interest as a neuroprotective agent in ICH because of its anti-inflammatory, antioxidant, and iron-chelating properties, with emerging preclinical evidence suggesting that these effects may also mitigate ferroptosis (Zhang R. et al., 2022). Accordingly, the Minocycline in Intracerebral Hemorrhage (MACH) trial, a pilot study, was conducted to evaluate the safety and efficacy of 400 mg minocycline over 5 days in ICH patients (Switzer, 2018) In this trial, they found 400 mg IV minocycline to be safe in ICH patients. Additionally, the pre-determined neuroprotective serum concentration was reached through IV administration (Fouda et al., 2017). Notably, despite MACH pilot study suggesting that IV administration of Minocycline achieved more rapid therapeutic concentration compared to oral dosing is acute setting, the ongoing phase III trial, MISTICH, is evaluating a 5-day oral regimen to evaluate the efficacy a and safety of 5-day Minocycline in acute ICH patients with 48 h of acute event (Zhao, 2026).

### Treatments via surgical/thrombolytic methods for blood extraction

Recent clinical trials for IVH have focused efforts in draining blood and thrombolysis of clots in the ventricles (Webb et al., 2012; Carrera et al., 2024; Hanley et al., 2017). While not directly targeting ferroptosis, these trials attempt to expedite blood removal, removing precursors required for ferroptosis (Tang et al., 2021). Clinical trials utilizing rt-PA to degrade clots have been shown to have dose–response effects on recovery by preventing blood clots, but the Phase III CLEAR trial did not show influence of primary endpoints compared to saline (Webb et al., 2012; Hanley et al., 2017). However, clinical data has suggested irrigating may also be beneficial by speeding blood removal in the ventricles, suggesting that gross blood removal from the ventricles may be a viable strategy (Hanley et al., 2017).

### Treatments targeting mitochondria

Compared to other forms of ICH, there is limited evidence for the mechanisms by which SDH induces neurodegenerative pathology from human studies, with most of what is known of SDH-induced neurodegeneration being discovered in preclinical animal models of SDH. One example of this is the mitochondrial dysfunction observed post-SDH. A retrospective study in humans demonstrated that patients with SDH developed mitochondrial dysfunction, specifically impaired aerobic metabolism, in the 24–48 h following the brain injury (Nordström et al., 2016). Additionally, human ultrastructural studies of cortical biopsies obtained during surgery for severe traumatic brain injury complicated by subdural hematoma have demonstrated swollen mitochondria and cristae disruption (Ishikawa et al., 2009), a hallmark of disrupted ionic and osmotic homeostasis. The mechanisms by which SDH induces this mitochondrial remodeling remains unclear, however research in other neurological injury models propose a potential explanation.

### Future treatments

No studies have yet demonstrated that selective inhibition of ferroptosis attenuates SDH or EDH pathology, and direct mechanistic evidence remains lacking. Future studies combining genetic manipulation with ferroptosis-specific inhibitors in animal models of SDH and EDH will be necessary to determine whether ferroptosis represents a primary driver of SDH/EDH pathology or a secondary consequence of iron-dependent oxidative stress (Table 2).

**Table 2:** Current evidence supporting iron accumulation, oxidative stress, mitochondrial dysfunction, and ferroptosis across intracranial hemorrhage subtypes.

Hemorrhage subtype	Iron overload	Oxidative stress	Mitochondrial dysfunction	Direct ferroptosis evidence
ICH	Strong	Strong	Strong	Strong
SAH	Strong	Strong	Moderate-Strong	Moderate
IVH	Moderate	Moderate	Limited	Limited
SDH	Limited	Limited	Moderate	Very Limited
EDH	Limited	Limited	Very limited	Absent

The strength of evidence reflects the quantity and consistency of available experimental and clinical studies and distinguishes well-established mechanisms from emerging or limited evidence.

## Conclusion

Intracranial hemorrhage leads to a complex series of secondary injuries that are strongly driven by iron released after blood breakdown. Across different types of intracranial bleeding, the accumulation of iron sets off oxidative reactions that damage lipids, proteins, and cellular membranes. This iron-dependent oxidative stress plays a central role in worsening neuronal injury beyond the initial bleed. Among the various cellular targets, mitochondria are especially vulnerable. Their disruption contributes to impaired energy production, increased ROS generation, and loss of normal cellular homeostasis, all of which further exacerbate neuronal dysfunction.

Ferroptosis has emerged as an important potential mechanism linking iron overload to cell death after intracranial hemorrhage. Many of the characteristic features of ferroptosis, such as iron-driven lipid peroxidation, depletion of antioxidant defenses, and distinct mitochondrial structural changes, have been observed in hemorrhagic injury models. These findings support the idea that ferroptosis can contributes to the progression of secondary brain injury. Identifying ferroptosis-related biochemical and mitochondrial biomarkers has also helped clarify how iron toxicity evolves over time and how it can play a role in ongoing neuronal damage.

Together, these insights highlight the importance of targeting iron accumulation, oxidative stress, and mitochondrial dysfunction as part of a broader strategy to reduce secondary injury after intracranial hemorrhage. Approaches such as iron chelation, inhibition of ferroptosis, and mitochondria-protective therapies have shown encouraging results in experimental models. Future work focused on optimizing these treatments and determining how they can be combined or applied clinically will be essential for improving outcomes in patients with ICH. Ultimately, targeting these pathways may provide a foundation for developing disease-modifying strategies aimed at mitigating long-term neurological impairment and potentially reducing the risk of post-stroke cognitive and motor decline.
